# Oxyhemoglobin Saturation Overshoot Following Obstructive Breathing Events Mitigates Sleep Apnea-Induced Glucose Elevations

**DOI:** 10.3389/fendo.2018.00477

**Published:** 2018-08-23

**Authors:** Luu V. Pham, Alan R. Schwartz, Jonathan C. Jun

**Affiliations:** Division of Pulmonary and Critical Care Medicine, Johns Hopkins University, Baltimore, MD, United States

**Keywords:** intermittent, hypoxia, metabolism, automated, phenotype

## Abstract

**Background:** Obstructive sleep apnea (OSA) and nocturnal hypoxia are associated with disturbances in glucose regulation and diabetes. Temporal associations between OSA, oxygenation profiles and glucose have not been well-described. We hypothesized that oxyhemoglobin desaturation during apneic events and subsequent post-apnea saturation overshoot predict nocturnal glucose.

**Methods:** In 30 OSA patients who underwent polysomnography while subjected to CPAP withdrawal, we characterized S_P_O_2_ swings by frequency, desaturation depth, and overshoot height relative to baseline. We examined the associations between frequently sampled glucose and S_P_O_2_ swings during the preceding 10 min. We developed multi-variable mixed effects linear regression to examine the independent associations between glucose and each level of these S_P_O_2_ swings, while controlling for OSA severity.

**Results:** Desaturation depth was not associated with glucose (*p* > 0.05). In contrast, overshoot was associated with glucose in a dose-dependent manner. Each S_P_O_2_ peak that did not rise to within 1% of baseline was associated with incremental glucose elevations of 0.49 mg/dL (*p* = 0.01), whereas peaks that exceeded baseline by >1% were associated with glucose reductions of 0.46 mg/dL. Overshoot remained an independent predictor of glucose after adjustment for mean S_P_O_2_ and OSA severity (*p* > 0.05).

**Conclusions:** Vigorous S_P_O_2_ improvements after apneic events may protect patients against OSA-related glucose elevations.

## Introduction

Obstructive sleep apnea (OSA) is a highly prevalent disease (1–3), which is associated with disturbances in glucose regulation including risks of type 2 diabetes (4–9). Intermittent closure of the upper airway in OSA causes hypoxia, sleep fragmentation and large intrathoracic pressure swings. Hypoxia may be an important stimulus for impaired glucose metabolism. In high altitude residents who are chronically exposed to ambient hypoxia, oxyhemoglobin saturation is associated with increased fasting glucose and glucose intolerance (10, 11). Investigators have also demonstrated that OSA-induced hypoxia was associated with glucose intolerance (7, 12). Furthermore, exposure to sustained or intermittent hypoxemia causes glucose intolerance and insulin resistance in human and animal experiments (13–16). In a recent study, we sampled blood at 20-min intervals during full polysomnography in CPAP-adherent OSA patients after discontinuing CPAP for 3 nights. During acute exposure to OSA, we observed that dynamic glucose elevations were closely preceded by periods of hypoxia, as assessed by the frequency of desaturations or median oxyhemoglobin saturation (S_P_O_2_) (17). These findings suggest rapid effects of OSA-related hypoxia on plasma glucose levels during sleep. Similarly, in a recent cross-sectional study in high altitude residents who are chronically exposed to hypoxia, we found that mean nocturnal S_P_O_2_ is associated with elevated hemoglobin A1c, independent of sleep apnea severity and daytime S_P_O_2_, indicating that nocturnal hypoxia contributes significantly to worsening overall glucose control (18). Nevertheless, the temporal associations between respiratory disturbances, oxygenation swings, and acute alterations in plasma glucose have not been well-described.

In OSA patients, the evolution of oxygenation over the course of the night is highly variable. During sleep, upper airway obstruction leads to falls in ventilation and subsequent oxyhemoglobin desaturation. At the termination of apneic events, arousals from sleep and improvements upper airway patency can result in transient increases in ventilation and oxygenation. The frequency and height of these S_P_O_2_ peaks can be influenced by several factors including ventilatory responses to gas exchange disturbances during apneic events (19), arousability, sympathetic activation, and co-morbid cardiopulmonary disease. These physiologic factors may not be fully reflected by traditional measures of sleep apnea and hypoxia severity including apnea hypopnea index (AHI) and time spent with S_P_O_2_ < 90% (T90). Therefore, dynamic oxygenation characteristics including frequency and depth of desaturation and subsequent correction may provide additional provide additional insight into metabolic risk in OSA patients.

We hypothesized that greater degrees of oxygen desaturation are associated with higher glucose, while greater oxygenation improvements after apneic events may protect against these elevations. To examine this hypothesis, we developed an automated approach to characterize oxygenation profiles in OSA patients by quantifying the frequency and height of periodic S_P_O_2_ nadirs and peaks. We then examined the associations between frequently sampled nocturnal plasma glucose levels and hypoxia, OSA severity, and dynamic S_P_O_2_ swings.

## Methods

The present study is a *post-hoc* analysis of a recent interventional trial of CPAP withdrawal. The recruitment, sleep study recording and blood sampling methods have been previously described (17). Briefly, CPAP-adherent patients with moderate-to-severe sleep apnea (AHI ≥ 20) underwent in-laboratory attended polysonography, with and without CPAP. During these studies, we measured glucose by sampling venous blood every 20 min. Because nocturnal glucose levels varied greatly and were distributed in a bimodal pattern between patients with and without diabetes, we limited our current analysis to non-diabetic patients. This study was approved by the Johns Hopkins Institutional Review Board, and all subjects provided informed written consent.

### Data analysis

#### Definitions

**Figure 1:**
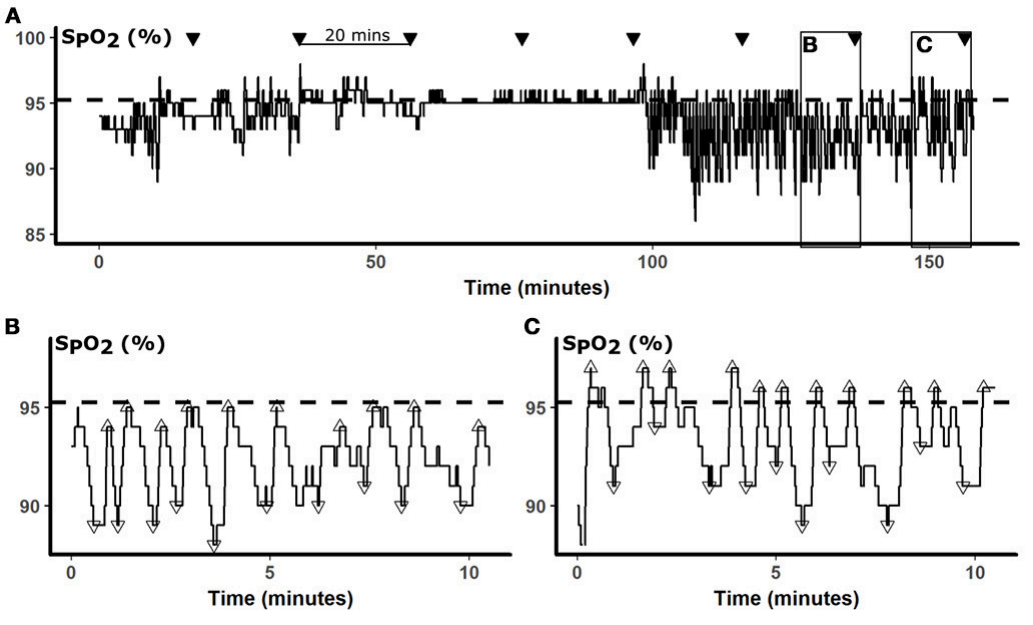
Data analysis. In **(A)**, a representative 160-min S_P_O_2_ is presented. The baseline S_P_O_2_ calculated from the mean S_P_O_2_ while treated with CPAP is represented by the dashed horizontal line. During sleep studies, glucose was measured every 20 min (▾). To examine S_P_O_2_ predictors of glucose, we analyzed oximetry during the 10 min preceding each glucose measurement, as illustrated by boxes in **(A)** and shown in greater details in **(B,C)**. S_P_O_2_ nadirs (▿) and peaks (Δ) were identified by automated peak detection. The desaturation depth and subsequent overshoot were calculated relative to the baseline. Panel **(B)** illustrates a period of frequent desaturations, which were followed by only partial restoration of oxygenation, resulting in negative overshoot. In contrast, Panel **(C)** illustrates repetitive desaturations were followed peaks that exceeded the baseline, resulting in positive S_P_O_2_ overshoots, despite desaturations were of similar depths.

Oxyhemogloblin saturation (S_P_O_2_) is dynamic and can vary about a baseline. Thus, we characterized oxygenation profiles by the frequency and amplitude of oxygen oscillations relative to baseline. Because the presence of sleep disordered breathing affects oxyhemoglobin saturation, we defined the baseline saturation as the mean S_P_O_2_ during treatment with CPAP, which resulted in stable respiratory patterns and oxygenation. This method also accounts for differences in lung function or co-morbidities that may also affect S_P_O_2_. Figure 1 illustrates the relationship between oxygenation in an OSA patient with repetitive apneic episodes, and the CPAP-treated baseline S_P_O_2_, which approximates oxygenation during stable breathing.

To detect acute changes in oxygenation, we applied an automated peak detection algorithm (Matlab, Natick, MA) to identify local S_P_O_2_ minima and maxima during the 10-min windows preceding blood draws (Figures 1B,C). Apneic events lead to falls in oxygenation and local oxygenation minima (▿). At the termination of apneas and hypopneas, increases in ventilation cause sharp rises in oxygen, resulting in local S_P_O_2_ maxima (Δ), that occasionally exceeded, or overshot, the baseline. Desaturation depths and overshoot heights of these minima and maxima, respectively, were calculated relative to the CPAP baseline. In Figure 1B, S_P_O_2_ fell from a baseline S_P_O_2_ of 95% to nadirs of between 89 and 91%, representing desaturation depths of 4–6%. Each of these desaturations was followed by incomplete correction of oxyhemoglobin saturation, to S_P_O_2_ levels of 1–2% below baseline (negative overshoot). In contrast, the frequent desaturations in Figure 1C were followed by over-correction of oxygen, exceeding the baseline by 1–3%. We analyzed our data in 10-min windows because sensitivity analyses demonstrated that model performance progressively decayed with increasing windows of 15 and 20 min. Additional sensitivity analyses were performed with progressively increasing lag times between S_P_O_2_ parameters and glucose.

#### Analytic methods

Our primary outcome was nocturnal glucose levels. Our repeated measures design allowed us to examine within- and between-subject changes in glucose, in association with variations in oxygenation and OSA severity. To minimize confounding from periods of wakefulness, we censored glucose measurements that were preceded by >30 s (5%) of wakefulness within 10 min of blood sampling. The 30-s threshold was chosen because blood sampling may occur in the middle of a 30-s scoring epoch, which would reduce the number epochs from 20 to 19. We compared glucose levels during periods with high and low overshoot heights and desaturation depths, using median overshoot (0.3%) and desaturation depth (5%) as the cutoffs. We stratified these analyses by periods with greater or less than the median number of apneic episodes in 10 min (7, which corresponds to an AHI of 42 episodes/h). In these analyses, we modeled glucose as a function of the density of apneic episodes (<7 vs. ≥7), high vs. low mean overshoot or desaturation depth, and their interaction with mixed-effects linear regression models. To further examine the association between nocturnal glucose levels and the frequency and degree of overshoot and desaturation, we developed univariable mixed-effects linear regression models of glucose at each point as functions of the number of overshoots of <–1, ≥–1 and <0, ≥0 and <1, and ≥1% during the preceding 10 min. Similar analyses were performed with desaturations at cutoffs of ≥4 and <5, ≥5 and <6, ≥6 and <7, and ≥7% cutoffs. To account for the effects of hypoxia on nocturnal plasma glucose, we developed multi-variable regression models to adjust for mean S_P_O_2_ preceding the glucose measurements. We then performed sensitivity analyses to examine the effect of including glucose measurements in the results. Data analyses were performed in R (www.r-project.org) with the “Linear Mixed-Effects Models using ‘Eigen' and S4” package.

## Results

### Subject baseline characteristics

Baseline subject characteristics are presented in Table 1. Thirty subjects met criteria for inclusion. The majority of subjects were male. On average, our subjects were middle aged and obese. OSA was present to a severe degree, with time with S_P_O_2_ < 90% for almost 20 min, on average.

**Table 1:** Demographic and polysomnographic characteristics.

**Characteristic**	**Mean (SD)**
*N*	30
Male sex [*n* (%)]	21 (70.0)
Observations per subject	10.77 (4.34)
Age (years)	48.83 (10.43)
BMI (kg/m^2^)	37.03 (7.88)
Sleep apnea severity (AHI) (events/h)	46.57 (29.18)
Time with S_P_O_2_ < 90 (minutes)	17.09 (19.13)
Mean S_P_O_2_ (%)	93.18 (2.01)

### Associations between desaturation depth and overshoot and plasma glucose during periods of high and low apnea density

**Figure 2:**
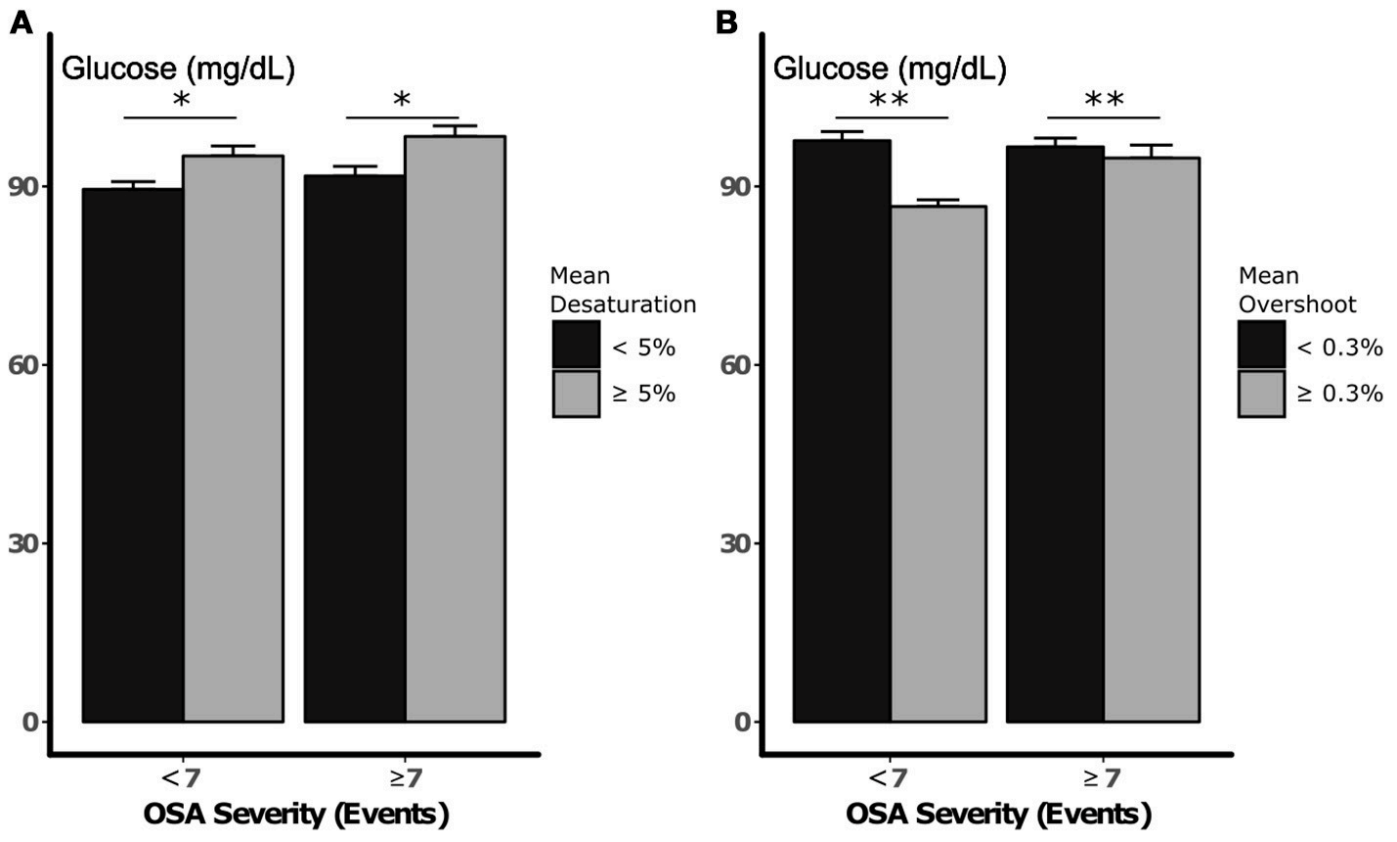
Nocturnal glucose vs. desaturation depth **(A)** and S_P_O_2_ overshoot **(B)**, during periods of low and high sleep apnea density. Error bars represent standard errors. *p < 0.10 and **p < 0.05, respectively, for independent association between mean desaturation depth and S_P_O_2_ and nocturnal glucose. Statistical analysis did not demonstrate an independent or interactive association between sleep apnea density and glucose during sleep.

Figure 2A illustrates glucose after periods of low and high densities of apneic events (greater than or less than 7 events per 10 min, respectively), with low and high mean desaturations. Glucose was higher after periods with greater falls in saturation regardless of event frequency (> 7 vs. < 7 apneas or hypopneas per period), although this difference did not exceed the threshold for statistical significance (*p* = 0.09). Neither independent association between sleep apnea density and glucose nor an interaction between desaturation and sleep apnea density was detected (*p* = 0.48 and 0.52, respectively). On the other hand, greater compared to less overshoot was independently associated with a 4.9 mg/dL reduction in glucose (*p* = 0.01, Figure 2B). In these analyses, an independent association between sleep apnea density and glucose was not observed, nor was there an interaction between sleep apnea density and S_P_O_2_ overshoot (*p* = 0.88 and 0.34, respectively). Sensitivity analysis with inclusion of periods of decreased sleep efficiency did not significantly alter these results.

### Independent associations between nocturnal glucose and desaturation and oxygenation overshoot frequency at varying levels of intensity

**Figure 3:**
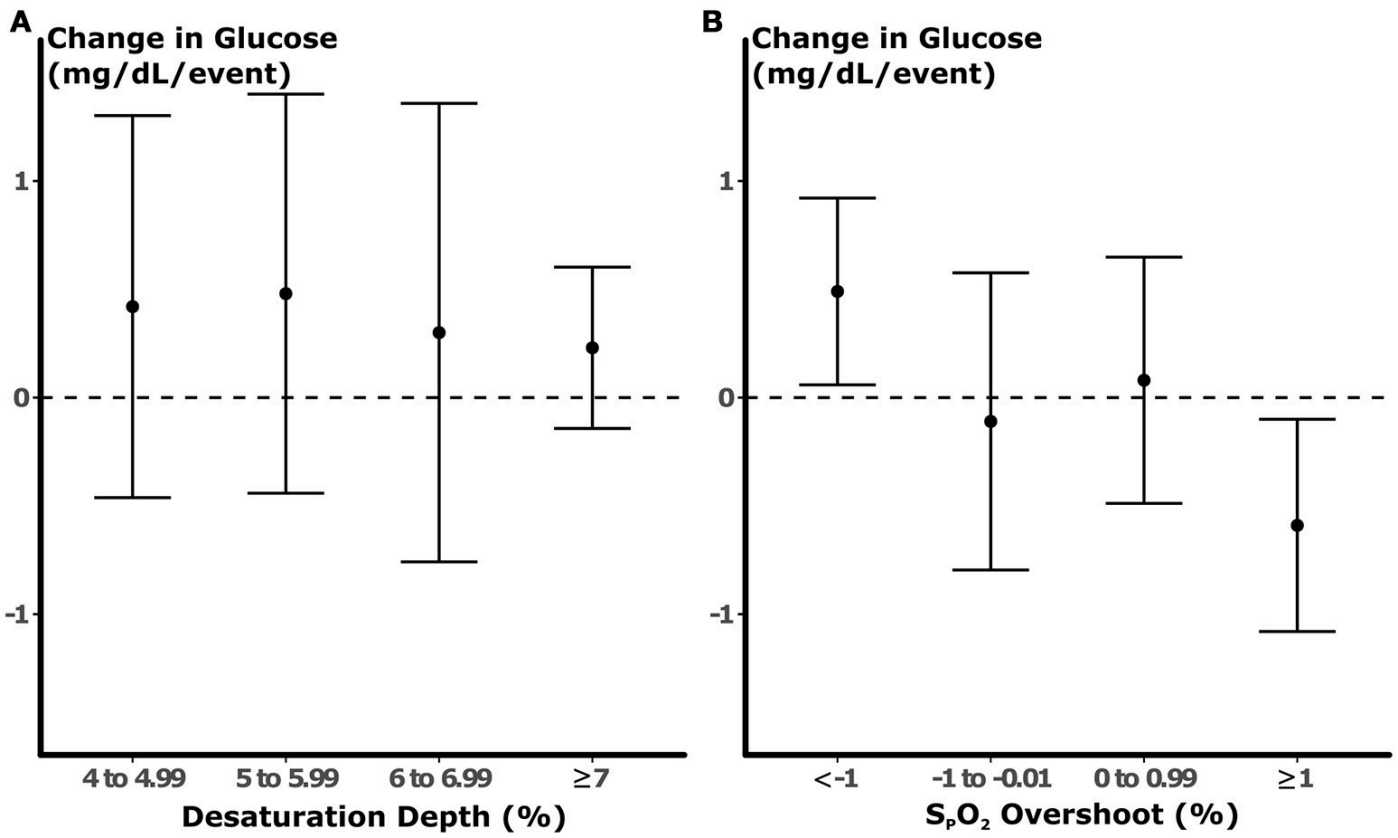
Results of univariable models of glucose. Each point represents the change in glucose (±95% confidence interval) predicted by the number of desaturation **(A)** or overshoot **(B)** during the preceding 10 min prior to blood sampling. No statistically significant association was observed between desaturation depth and glucose. In contrast, each apneic episode that was followed by partial normalization of oxygenation was associated with an increase in glucose by 0.49 mg/dL. With increasing degrees of S_P_O_2_ overshoot, the changes in glucose related to each event fell, and S_P_O_2_ peaks that exceeded baseline by >1% were associated with reductions in glucose.

The association between desaturation frequency at varying levels and nocturnal glucose is illustrated in Figure 3A. We did not observe a significant association between the depth of desaturation and glucose. Nevertheless, all beta coefficients were similar and approached the threshold for significance (*p* = 0.07) when all desaturation levels were pooled prior to analysis (see $supplement). In contrast, overshoot height predicted glucose in a dose-dependent manner (Figure 3B). At negative levels of S_P_O_2_ overshoot (i.e., when S_P_O_2_ did not return to within 1% of the baseline after an apneic episode), each S_P_O_2_ peak during the preceding 10 min was associated with an incremental increase in glucose of 0.49 mg/dL. With greater degrees of overshoot, the incremental change in glucose associated with each apneic event progressively fell. In fact, vigorous overshoots that exceeded baseline saturation by ≥1% were associated with reductions in glucose of 0.46 mg/dL (*p* = 0.03). In multi-variable models that incorporated all levels of S_P_O_2_ overshoot, very low levels of overshoot (<–1%) were independently associated with increases in glucose (Table 2). In these models, we observed a trend between ≥1% overshoot above the baseline and reductions in glucose (*p* = 0.07). The associations between ≥1% overshoot and lower glucose was significant after adjustment for traditional metrics of sleep-disordered breathing, including sleep disordered breathing frequency and mean S_P_O_2_. The associations between partial oxygenation correction (<–1% overshoot) with increased glucose was not significant with these adjustments because of collinearity with mean S_P_O_2_. These findings suggest that the degree of oxygenation overshoot, but not desaturation depth, after apneic events is a robust predictor of nocturnal glucose.

**Table 2:** Regression models of associations between plasma glucose and S_P_O_2_ overshoot and sleep disordered breathing severity.

	**Uni-variable models**		**Multi-variable model 1**		**Multi-variable model 2**	
**Predictor**	**Beta**	***p*-value**	**Beta**	***p*-value**	**Beta**	***p*-value**
S_P_O_2_ Overshoot						
< –1%	0.49	0.01	0.42	0.04	0.24	0.33
−1 to (−0.01)%	−0.11	0.77	−0.01	0.67	–	–
0 – 0.99%	−0.14	0.54	−0.00	0.99	–	–
≥1%	−0.46	0.03	−0.40	0.07	−0.46	< 0.05
Mean S_P_O_2_	−0.89	0.005	–	–	−0.63	0.08
Apneas and hypopneas	0.10	0.36	–	–	0.10	0.40

## Discussion

In the present study, we examined OSA-related hypoxia profiles and their association with glucose during sleep. The novel finding of the study is that the pattern of hypoxia is an important predictor of the overall effect of hypoxia on nocturnal glucose level. Greater S_P_O_2_ overshoot was dose-dependently associated with lower glucose, independent of mean S_P_O_2_ or the frequency of sleep apnea events. As expected, acute hypoxia was also dynamically associated with elevated plasma glucose. Interestingly, there was no relationship between the frequency of OSA events or desaturation depth after these events, and nocturnal plasma glucose levels. Taken together, OSA characterized by nocturnal hypoxia and infrequent and incomplete restoration of oxygenation reflects a phenotype that may be more vulnerable to nocturnal glucose elevation. Conversely, large S_P_O_2_ overshoot may blunt the impact of sleep disordered breathing on nocturnal glucose in OSA patients.

Several mechanisms may explain the links between oxygenation profiles and OSA-related glucose disturbances. First, episodic oxygen restoration may mitigate nocturnal hypoxia related to apneic events. Frequent intermittent oxygen rises may be indicative of frequent arousals, which shorten apnea and hypopnea duration (20–22). In addition, large inspiratory efforts preceding brisk S_P_O_2_ rises could also increase oxygen stores in the lungs and diminish subsequent desaturations. Second, vigorous overshoot could be a marker of arousal and/or lighter sleep, which limits the likelihood of developing additional apneic events and metabolic disturbances (23). In fact, the association between S_P_O_2_ overshoot of >1% and glucose lowering did not attenuate with adjustment for mean nocturnal S_P_O_2_, suggesting that S_P_O_2_ overshoot may reduce plasma glucose through mechanisms independent of hypoxia. Alternatively, the degree of hypoxia during apneic events and subsequent overshoot could be influenced by comorbidities (24–28). For example, patients with visceral obesity or cardio-pulmonary dysfunction may exhibit both blunted S_P_O_2_ rises and higher glucose excursions after apneic episodes.

In contrast to S_P_O_2_ overshoot, desaturation depth was not related to the degree of glucose change. Further analyses with commonly clinically reported metrics of hypoxia severity including time with S_P_O_2_ < 90% also did not predict nocturnal glucose (data not shown). The present study also did not demonstrate an association between OSA events and dynamic nocturnal glucose changes. These findings imply that existing metrics of OSA severity and related hypoxia do not adequately predict the glycemic impact of sleep disordered breathing. In contrast to previous approaches to characterizing nocturnal hypoxia, we implemented a readily deployable, automated algorithm that focuses on a novel dimension of the oxygenation profile that captures the additional physiologic responses to sleep disordered breathing. Our study demonstrates that frequency and degree of oxygenation overshoots is an important predictor of OSA-related metabolic sequelae, independent of traditional measures of sleep apnea severity.

Our study has several limitations, which are worth considering when interpreting the results. First, the observational nature of our study limits causal inferences. Nevertheless, we demonstrated that alterations in mean S_P_O_2_ and S_P_O_2_ overshoot occurred before changes in nocturnal glucose. Moreover, S_P_O_2_ overshoot predicted nocturnal glucose changes in a dose-dependent manner. These findings are consistent with the notion that hypoxia causes glucose elevations, which can be mitigated by intermittent improvements in oxygenation. Second, our method defines baseline S_P_O_2_ from polysonography while treated with CPAP. The requirement for stable respiratory patterns on a separate night may limit the ability to deploy our methods to estimate metabolic risk in a clinical setting. CPAP may also increase lung volumes and improve baseline S_P_O_2_ (29). Nevertheless, low lung volumes and attendant S_P_O_2_ reductions may be part of the causal pathway in the pathogenesis of OSA-related hyperglycemia. Third, we examined the relationship between oxygenation and glucose profiles in non-diabetic patients with moderate-to-severe OSA. Additional studies are required to determine the generalizability of these methods to patients with diabetes and/or mild disease by AHI criteria.

## Conclusions

In summary, our study demonstrated that intermittent restoration of S_P_O_2_ was independently associated with dynamic nocturnal glucose reductions. In contrast, reductions in mean S_P_O_2_ but not the nadir S_P_O_2_ after apneic events predicted glucose elevations. These findings indicate that nocturnal hypoxia is an important determinant of OSA-related glucose disturbances. Our findings further imply that interventions that improve oxygenation in OSA patients may mitigate nocturnal glucose excursions. Additional studies are required to examine the role of oxygen to prevent worsening glucose control, and prospectively validate the use of oxygenation profile analysis to predict metabolic risk in OSA.

## Author contributions

LP, AS, and JJ contributed to the data analysis and interpretation, and manuscript preparation.

### Conflict of interest statement

The authors declare that the research was conducted in the absence of any commercial or financial relationships that could be construed as a potential conflict of interest.

## References

[1] Young T Palta M Dempsey J Skatrud J Weber S Badr S. The occurrence of sleep-disordered breathing among middle-aged adults. N Engl J Med. (1993) 328:1230–5. 10.1056/NEJM199304293281704 8464434

[2] Peppard PE Young T Barnet JH Palta M Hagen EW Hla KM. Increased prevalence of sleep-disordered breathing in adults. Am J Epidemiol. (2013) 177:1006–14. 10.1093/aje/kws342 23589584 PMC3639722

[3] Heinzer R Vat S Marques-Vidal P Marti-Soler H Andries D Tobback N. Prevalence of sleep-disordered breathing in the general population: the HypnoLaus study. Lancet Respir Med. (2015) 3:310–8. 10.1016/S2213-2600(15)00043-0 25682233 PMC4404207

[4] Ip MS Lam B Ng MM Lam WK Tsang KW Lam KS. Obstructive sleep apnea is independently associated with insulin resistance. Am J Respir Crit Care Med. (2002) 165:670–6. 10.1164/ajrccm.165.5.2103001 11874812

[5] Punjabi NM Beamer BA. Alterations in glucose disposal in sleep-disordered breathing. Am J Respir Crit Care Med. (2009) 179:235–40. 10.1164/rccm.200809-1392OC 19011148 PMC2633056

[6] Reichmuth KJ Austin D Skatrud JB Young T. Association of sleep apnea and type II diabetes: a population-based study. Am J Respir Crit Care Med. (2005) 172:1590–5. 10.1164/rccm.200504-637OC 16192452 PMC2718458

[7] Punjabi NM Shahar E Redline S Gottlieb DJ Givelber R Resnick HE. Sleep-disordered breathing, glucose intolerance, and insulin resistance: the sleep heart health study. Am J Epidemiol. (2004) 160:521–30. 10.1093/aje/kwh261 15353412

[8] Bakker JP Weng J Wang R Redline S Punjabi NM Patel SR. Associations between Obstructive sleep apnea, sleep duration, and abnormal fasting glucose. The multi-ethnic study of atherosclerosis. Am J Respir Crit Care Med. (2015) 192:745–53. 10.1164/rccm.201502-0366OC 26084035 PMC4595677

[9] Nagayoshi M Punjabi NM Selvin E Pankow JS Shahar E Iso H. Obstructive sleep apnea and incident type 2 diabetes. Sleep Med. (2016) 25:156–61. 10.1016/j.sleep.2016.05.009 27810258 PMC5102826

[10] Okumiya K Sakamoto R Ishimoto Y Kimura Y Fukutomi E Ishikawa M. Glucose intolerance associated with hypoxia in people living at high altitudes in the Tibetan highland. BMJ Open (2016) 6:e009728. 10.1136/bmjopen-2015-009728 26908520 PMC4769430

[11] Miele CH Schwartz AR Gilman RH Pham L Wise RA Davila-Roman VG. Increased cardiometabolic risk and worsening hypoxemia at high altitude. High Alt Med Biol. (2016) 17:93–100. 10.1089/ham.2015.0084 27281472 PMC4913510

[12] Punjabi NM Sorkin JD Katzel LI Goldberg AP Schwartz AR Smith PL. Sleep-disordered breathing and insulin resistance in middle-aged and overweight men. Am J Respir Crit Care Med. (2002) 165:677–82. 10.1164/ajrccm.165.5.2104087 11874813

[13] Mesarwi O Sharma EV Jun J Polotsky VY. Metabolic dysfunction in obstructive sleep apnea: a critical examination of underlying mechanisms. Sleep Biol Rhythms (2015) 13:2–17. 10.1111/sbr.12078 26412981 PMC4583137

[14] Louis M Punjabi NM. Effects of acute intermittent hypoxia on glucose metabolism in awake healthy volunteers. J Appl Physiol. (2009) 106:1538–44. 10.1152/japplphysiol.91523.2008 19265062 PMC2681331

[15] Oltmanns KM Gehring H Rudolf S Schultes B Rook S Schweiger U. Hypoxia causes glucose intolerance in humans. Am J Respir Crit Care Med. (2004) 169:1231–7. 10.1164/rccm.200308-1200OC 15044204

[16] Newhouse LP Joyner MJ Curry TB Laurenti MC Man CD Cobelli C. Three hours of intermittent hypoxia increases circulating glucose levels in healthy adults. Physiol Rep. (2017) 5:e13106. 10.14814/phy2.13106 28087818 PMC5256164

[17] Chopra S Rathore A Younas H Pham LV Gu C Beselman A. Obstructive sleep apnea dynamically increases nocturnal plasma free fatty acids, glucose, and cortisol during sleep. J Clin Endocrinol Metab. (2017) 102:3172–81. 10.1210/jc.2017-00619 28595341 PMC5587067

[18] Pham LV Miele CH Schwartz NG Arias RS Rattner A Gilman RH. Cardiometabolic correlates of sleep disordered breathing in Andean highlanders. Eur Respir J. (2017) 49:1601705. 10.1183/13993003.01705-2016 28619952 PMC5765983

[19] Younes M Ostrowski M Thompson W Leslie C Shewchuk W. Chemical control stability in patients with obstructive sleep apnea. Am J Respir Crit Care Med. (2001) 163:1181–90. 10.1164/ajrccm.163.5.2007013 11316657

[20] Findley LJ Wilhoit SC Suratt PM. Apnea duration and hypoxemia during REM sleep in patients with obstructive sleep apnea. Chest (1985) 87:432–6. 10.1378/chest.87.4.432 3979129

[21] Basner RC Onal E Carley DW Stepanski EJ Lopata M. Effect of induced transient arousal on obstructive apnea duration. J Appl Physiol. (1995) 78:1469–76. 10.1152/jappl.1995.78.4.1469 7615457

[22] Amatoury J Azarbarzin A Younes M Jordan AS Wellman A Eckert DJ. Arousal intensity is a distinct pathophysiological trait in obstructive sleep apnea. Sleep (2016) 39:2091–100. 10.5665/sleep.6304 27784404 PMC5103797

[23] Longobardo GS Evangelisti CJ Cherniack NS. Analysis of the interplay between neurochemical control of respiration and upper airway mechanics producing upper airway obstruction during sleep in humans. Exp Physiol. (2008) 93:271–87. 10.1113/expphysiol.2007.039917 17933858

[24] Schafer H Pauleit D Sudhop T Gouni-Berthold I Ewig S Berthold HK. Body fat distribution, serum leptin, and cardiovascular risk factors in men with obstructive sleep apnea. Chest (2002) 122:829–39. 10.1378/chest.122.3.829 12226021

[25] Zavorsky GS Hoffman SL. Pulmonary gas exchange in the morbidly obese. Obes Rev. (2008) 9:326–39. 10.1111/j.1467-789X.2008.00471.x 18331421

[26] Borel AL Monneret D Tamisier R Baguet JP Faure P Levy P. The severity of nocturnal hypoxia but not abdominal adiposity is associated with insulin resistance in non-obese men with sleep apnea. PLoS ONE (2013) 8:e71000. 10.1371/journal.pone.0071000 23951064 PMC3741390

[27] Vos PJ Folgering HT van Herwaarden C. Predictors for nocturnal hypoxaemia (mean SaO2 < 90%) in normoxic and mildly hypoxic patients with COPD. Eur Respir J. (1995) 8:74–7. 10.1183/09031936.95.08010074 7744197

[28] McNicholas WT Verbraecken J Marin JM. Sleep disorders in COPD: the forgotten dimension. Eur Respir Rev. (2013) 22:365–75. 10.1183/09059180.00003213 23997063 PMC9487346

[29] Findley LJ Ries AL Tisi GM Wagner PD. Hypoxemia during apnea in normal subjects: mechanisms and impact of lung volume. J Appl Physiol. (1983) 55:1777–83. 10.1152/jappl.1983.55.6.1777 6662768

